# Development and validation of a prognostic nomogram for predicting hypostatic pneumonia risk in large vessel occlusion stroke after endovascular therapy patients

**DOI:** 10.3389/fneur.2025.1654147

**Published:** 2026-01-07

**Authors:** Jingling Zhu, Wenfei Liang, Yu Ding, Xiaohua He, Jiasheng Zhao, Guoshun Li, Zhaobang Chen, Kangqiang Yang, Xiaoling Wu, Bin Liao, Huiquan Deng, Zichong Liang, Zhan Zhao, Jingyi Chen, Qiuxing He, Weimin Ning

**Affiliations:** 1 Department of Neurology, Dongguan Hospital of Guangzhou University of Chinese Medicine, Dongguan, China; 2 Dongguan Key Laboratory of Intractable Brain Diseases in Dongguan, Dongguan Hospital of Guangzhou University of Chinese Medicine, Dongguan, China; 3 State Key Laboratory of Dampness Syndrome of Chinese Medicine, Dongguan Hospital of Guangzhou University of Chinese Medicine, Dongguan, China

**Keywords:** acute ischemic stroke with large vessel occlusion, endovascular therapy, hypostatic pneumonia, nomogram, postoperative complications

## Abstract

**Background:**

Post-stroke hypostatic pneumonia (HP) significantly impairs neurological recovery and worsens prognosis in patients with acute ischemic stroke with large vessel occlusion (AIS-LVO). This study aimed to develop and validate a prognostic nomogram for predicting hypostatic pneumonia risk following endovascular therapy (EVT) in AIS-LVO patients.

**Methods:**

We retrospectively analyzed 650 consecutive AIS-LVO patients who underwent endovascular therapy with mechanical ventilation at Dongguan Hospital of Guangzhou University of Chinese Medicine from September 2018 to March 2025. After applying inclusion/exclusion criteria, 412 patients were randomly split into two groups: training (*n* = 288) and validation (*n* = 124), maintaining a 7:3 ratio. Using least absolute shrinkage and selection operator (LASSO) regression for feature selection followed by multivariable logistic regression, we identified independent predictors for nomogram construction. Model performance was assessed through the receiver operating characteristic curve (ROC), calibration curve, decision curve analysis (DCA), and clinical impact curve (CIC).

**Results:**

Four independent predictors were identified: admission Glasgow Coma Scale (GCS) score (OR 0.77, 95% CI 0.68–0.86), postoperative 48 h fever (OR 2.77, 95% CI 1.52–5.02), postoperative 48 h neutrophil-to-lymphocyte ratio (NLR) (OR 1.15, 95% CI 1.08–1.22), and ASPECTS (OR 0.74, 95% CI 0.63–0.87). The model had an area under the curve (AUC) of 0.829 (95% CI: 0.781–0.877) in the training cohort and 0.817 (95% CI 0.732–0.903) in the validation cohort, which means it was good at making predictions. Calibration curves revealed good alignment between predicted and observed probabilities in the training cohort. The validation cohort retained satisfactory calibration, with only modest overestimation of risk. DCA and CIC consistently indicated the nomogram’s applicability in diverse clinical settings.

**Conclusion:**

We developed and validated an effective nomogram incorporating four clinically accessible parameters to predict the risk of hypostatic pneumonia after EVT. This tool may facilitate early high-risk patient identification and guide preventive therapy to improve clinical outcomes.

## 1 Introduction

Stroke remains the second leading cause of global mortality, with ischemic stroke constituting the predominant subtype and imposing substantial socioeconomic burdens through long-term disability (1). While endovascular therapy (EVT) has significantly improved revascularization rates and functional prognosis in patients with acute ischemic stroke with large vessel occlusion (AIS-LVO) (2–5), post-procedural complications, particularly pneumonia, continue to compromise clinical benefits. Pneumonia complicates the hospitalization of 40% of critically ill acute stroke patients (6), and it is associated with exacerbated neurological injury mediated by systemic inflammation (7). Importantly, pneumonia may precipitate life-threatening complications including sepsis, multi-organ dysfunction, and death (7).

Among various pneumonia subtypes, hypostatic pneumonia (HP) represents a distinct clinical entity characterized by its unique pathophysiology and diagnostic challenges. HP develops due to prolonged immobilization, leading to gravitational pulmonary congestion, impaired mucociliary clearance, and subsequent bacterial colonization in dependent lung regions (8, 9). This condition is particularly prevalent in neurologically compromised populations, including post-stroke patients and elderly individuals with limited mobility (10). However, current diagnostic paradigms frequently fail to distinguish HP from other forms of pneumonia due to overlapping clinical features and nonspecific diagnostic criteria (e.g., radiographic infiltrates accompanied by fever, elevated or decreased leucocyte count) (8, 11). This diagnostic ambiguity often results in delayed recognition and suboptimal management.

The development of accurate prediction tools for post-EVT hypostatic pneumonia is further complicated by several knowledge gaps in existing literature. First, most studies have treated pneumonia as a homogeneous entity without accounting for subtype-specific risk factors. Second, no dedicated prediction models exist for hypostatic pneumonia. Current predictive tools primarily target general post-stroke pneumonia and lack validation for this distinct subtype. Third, methodological limitations including small sample sizes and inadequate variable selection techniques have constrained model performance.

To address these limitations, we developed and validated a novel nomogram incorporating both established clinical predictors and innovative inflammatory indices. Our approach features three key advancements: (1) explicit focus on hypostatic pneumonia as a distinct clinical endpoint, recognizing the synergistic effects of neurological impairment and post-procedural factors; (2) systematic integration of novel inflammatory biomarkers including neutrophil-to-lymphocyte ratio (NLR), systemic immune-inflammation index (SII), platelet-to-lymphocyte ratio (PLR), and systemic inflammatory response index (SIRI), with variable selection optimized through LASSO regression to mitigate multicollinearity; (3) nomogram development using a comparatively large, well-characterized cohort of EVT-treated AIS-LVO patients.

This study represents a significant step toward personalized risk assessment for post-EVT hypostatic pneumonia. By enabling early identification of high-risk patients, our nomogram may facilitate timely implementation of preventive strategies such as targeted respiratory therapy or prophylactic antibiotics, ultimately reducing pneumonia-related morbidity and mortality in this vulnerable population.

## 2 Methods

### 2.1 Study design and patient selection

We performed a retrospective analysis of patients with AIS-LVO who received EVT at Dongguan Hospital of Guangzhou University of Chinese Medicine (September 2018 to March 2025). The inclusion criteria were as follows: (1) confirmed LVO (internal carotid artery, M1/M2 middle cerebral artery, or basilar artery) by computed tomography angiography (CTA), magnetic resonance angiography (MRA), or digital subtraction angiography (DSA); (2) AIS diagnosis according to World Health Organization criteria with neuroimaging confirmation (computed tomography [CT] or magnetic resonance imaging [MRI]); (3) age ≥18 years; Exclusion criteria were: (1) pre-existing pulmonary or systemic infection (confirmed by microbiological or radiographic evidence); (2) severe comorbidities (e.g., end-stage organ failure, hematologic disorders); (3) immunocompromised status (HIV, immunosuppressant use, etc.); (4) modified Thrombolysis in Cerebral Infarction (mTICI) grade ≤ 2a without recanalization after EVT; (5) incomplete clinical data or loss to follow-up. After screening 650 patients, 412 met eligibility criteria and were subsequently allocated into a training cohort (*n* = 288) and a validation cohort (*n* = 124) in a 7:3 ratio. This research was approved by the Institutional Review Board of Dongguan Hospital (No. PJ[2025]88), with informed consent waived due to the retrospective use of anonymized data.

### 2.2 Outcome definition: hypostatic pneumonia

The HP was diagnosed within 7 days after EVT by two independent neurologists blinded to the predictors; any diagnostic discrepancies were arbitrated by a senior neurologist experienced in managing stroke complications. The diagnosis required simultaneous fulfillment of the following three criteria (8, 12, 13): (1) Clinical manifestations: ≥2 of cough, fever (>38 °C), purulent sputum, and deteriorated respiratory status, coupled with prolonged bed rest and reduced or absent sputum excretion; (2) Laboratory findings: Peripheral white blood cell (WBC) count >10 × 10^9^/L or <4 × 10^9^/L; (3) Radiographic evidence: Chest X-ray showing irregular, tiny patchy high-density shadows (with blurred edges and uneven density) in the lower lung fields.

Given the lack of unified diagnostic guidelines for HP, this study established the diagnostic framework and observation window with reference to key literature (8, 12, 13): a comprehensive approach combining clinical, radiological, and laboratory assessments was adopted, along with a 7-day observation window. This window reflects how HP develops—specifically, HP is a secondary infection driven by pulmonary secretion accumulation from post-EVT immobilization.

### 2.3 Predictor variables and data collection

We identified 28 predictor variables for HP in EVT patients through literature retrieval, and these variables were then systematically extracted from electronic medical records. Meanwhile, multicollinearity tests were performed on these variables using SPSS software. The results showed that the variance inflation factor (VIF) of all variables was less than 10 (Supplementary Material 2), indicating no significant multicollinearity among the variables. These variables included: demographics (age, gender), medical history (hypertension, diabetes, atrial fibrillation, prior stroke, smoking, drinking), clinical status [dysphagia, postoperative 48 h fever, admission sbp, admission dbp, admission NIHSS (14), admission GCS, admission mRS (1, 15)], laboratory indicators [postoperative 24 h CRP, postoperative 24 h NLR (16), postoperative 24 h SII, postoperative 24 h SIRI (17), postoperative 24 h PLR, postoperative 24 h TyG, postoperative 24 h SHR], imaging/etiologic data [ASPECTS (18, 19), TOAST classification, fazekas scale, infarction site], and procedure-related parameters (operation time, symptom onset to puncture time).

### 2.4 Statistical analysis

The SPSS software (version 27.0), R software (version 4.2.3), and Origin 2024 were the primary tools utilized for data analysis and visualization. To reduce selection bias and ensure baseline balance between the training and validation cohorts, propensity score matching (PSM) was first conducted to adjust for confounders. Descriptive statistical analysis was then performed for 412 participants after PSM.

Continuous variables were presented as mean ± SD (normal distribution, independent samples *t*-test) or median (IQR) (non-normal distribution, Mann–Whitney *U* test). Categorical variables were reported as frequencies (%) and compared via chi-square test (expected frequency ≥5) or Fisher’s exact test (expected frequency <5). Two-sided *p* < 0.05 was statistically significant.

Missing values were present only for the stress hyperglycemia ratio (SHR) (21/412 cases, 5.10%), resulting in an overall missing rate <10%. Following confirmation of missing at random (MAR), multiple imputation (SPSS, 5 iterations) was used for handling. Sensitivity analysis confirmed model robustness, with consistent AUC (0.827 vs. 0.824) and no substantial differences in key metrics. Detailed comparisons are available in Supplementary Material 1.

Sample size was estimated using the Events Per Variable (EPV) principle for binary logistic regression-based predictive models. To construct a nomogram predicting HP in AIS-LVO patients after EVT (HP as the outcome), key parameters were set as follows: 4–6 expected independent variables, a minimum EPV of 10, an anticipated HP incidence of 35%, and a 70% training set proportion. This yielded a minimum required training set size of ~171.4 cases, translating to a total sample size of ~245 cases (before accounting for invalid samples). Considering a 10% reserve for invalid data, the final minimum total sample size was 273 cases. A total of 412 eligible patients were enrolled, meeting the sample size requirement.

Multicollinearity among all candidate predictor variables was diagnosed using the VIF. LASSO regression was applied for feature selection, with the optimal penalty parameter (lambda, *λ*₁se = 0.053) determined as the value that corresponds to the cross-validation error within one standard deviation of the minimum error. The variables selected by LASSO were incorporated into a multivariable logistic regression model, and multicollinearity was re-evaluated to ensure the stability of parameter estimates. For model performance assessment, discrimination was evaluated using the Receiver Operating Characteristic (ROC) curve and the Area Under the Curve (AUC); goodness-of-fit and calibration were comprehensively assessed via the Hosmer-Lemeshow test, calibration curves, and Brier score; additionally, Decision Curve Analysis (DCA) and Clinical Impact Curve (CIC) were utilized to evaluate the clinical net benefit across different decision thresholds, thereby verifying the model’s clinical utility.

## 3 Results

### 3.1 Study flow diagram

**Figure 1:**
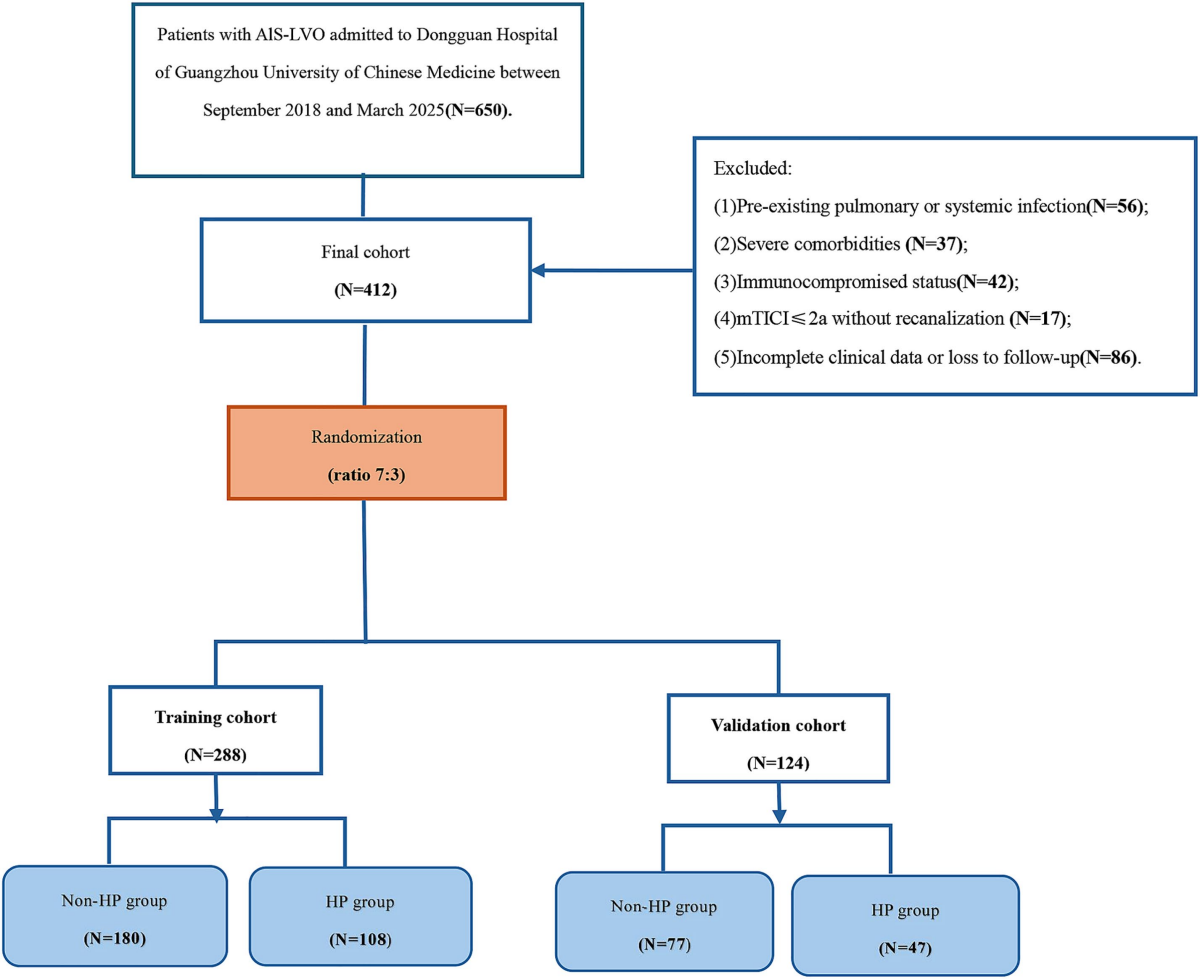
Study design flowchart.

Figure 1 presents the participant selection flowchart. From an initial cohort of 650 patients with AIS-LVO who underwent EVT, 238 were excluded based on predefined criteria (detailed in Methods). The final analytical cohort comprised 412 patients, who were allocated to either the training cohort (*n* = 288) or validation cohort (*n* = 124) in a 7:3 ratio.

### 3.2 Patient characteristics

To eliminate potential selection bias and ensure the comparability of baseline data between the training and validation cohorts, we first applied propensity score matching (PSM) to adjust for confounding factors. A total of 412 patients were recruited for this study, including 257 patients without HP (62.38%) and 155 patients with HP (37.62%). After PSM, the 412 patients were allocated into a training cohort (*n* = 288) and a validation cohort (*n* = 124) at a 7:3 ratio to ensure the reliability of subsequent model development and validation.

Table 1 presents the baseline characteristics of the two cohorts, including demographic data, clinical scores, laboratory indices, procedure-related variables, medical history, and clinical status. The “Statistic” and “*P*” columns show the results of differential analysis between the two cohorts. No statistically significant differences were observed in any variable (all *p* > 0.05), indicating that the training and validation cohorts were balanced and comparable, which guaranteed the validity of cross-validation for the prediction model.

**Table 1:** A comparison of the baseline characteristics between the training and validation cohorts (after propensity score matching).

Variable	Total (*n* = 412)	Training cohort (*n* = 288)	Validation cohort (*n* = 124)	Statistic	*p-*value
Hypostatic pneumonia, *n* (%)				*χ*^2^ = 0.006	0.938
No	257 (62.38)	180 (62.50)	77 (62.10)		
Yes	155 (37.62)	108 (37.50)	47 (37.90)		
Age, *M* (Q₁, Q₃)	60.50 (51.00, 73.00)	60.00 (51.00, 73.00)	61.50 (49.75, 75.00)	*Z* = −0.307	0.759
Admission GCS, *M* (Q₁, Q₃)	15.00 (13.00, 15.00)	15.00 (13.00, 15.00)	15.00 (12.00, 15.00)	*Z* = −0.932	0.351
Admission NIHSS, *M* (Q₁, Q₃)	8.00 (4.00, 13.00)	8.00 (4.00, 13.00)	10.00 (5.00, 15.00)	*Z* = −1.567	0.117
Admission ASPECTS, *M* (Q_1_, Q_3_)	8.00 (6.75, 8.00)	8.00 (7.00, 8.00)	8.00 (6.00, 8.00)	*Z* = −0.059	0.953
Admission SBP, *M* (Q₁, Q₃)	150.00 (133.75, 166.00)	150.00 (133.00, 165.00)	146.00 (136.00, 166.00)	*Z* = −0.335	0.738
Admission DBP, *M* (Q₁, Q₃)	88.00 (79.00, 98.00)	89.00 (79.00, 98.25)	86.00 (78.00, 96.00)	*Z* = −0.784	0.433
Postoperative 24 h SHR, *M* (Q₁, Q₃)	1.03 (0.87, 1.26)	1.04 (0.87, 1.28)	1.01 (0.85, 1.20)	*Z* = −1.426	0.154
Postoperative 24 h TyG, *M* (Q₁, Q₃)	9.61 (9.24, 10.08)	9.62 (9.24, 10.10)	9.59 (9.27, 9.96)	*Z* = −0.888	0.375
Postoperative 24 h CRP, *M* (Q₁, Q₃)	4.40 (2.05, 12.59)	4.19 (2.04, 11.40)	5.30 (2.10, 12.79)	*Z* = −0.967	0.334
Postoperative 24 h SII, *M* (Q₁, Q₃)	1226.29 (809.86, 2190.32)	1273.34 (844.80, 2179.22)	1088.78 (747.49, 2214.91)	*Z* = −1.465	0.143
Postoperative 24 h SIRI, *M* (Q₁, Q₃)	2.11 (1.26, 3.85)	2.10 (1.18, 3.73)	2.11 (1.36, 4.04)	*Z* = −0.994	0.320
Postoperative 24 h NLR, *M* (Q₁, Q₃)	5.57 (3.55, 9.77)	5.70 (3.52, 9.77)	5.26 (3.64, 9.57)	*Z* = −0.428	0.669
Postoperative 24 h PLR, *M* (Q₁, Q₃)	164.87 (111.39, 249.04)	168.54 (118.90, 255.35)	158.21 (103.63, 242.30)	*Z* = −1.446	0.148
Operation time (the interval from groin puncture to successful reperfusion), *M* (Q_1_, Q_3_)	0.90 (0.73, 1.11)	0.90 (0.73, 1.06)	0.90 (0.71, 1.11)	*Z* = −0.072	0.943
Symptom onset to puncture, *M* (Q_1_, Q_3_)	5.25 (3.57, 8.35)	5.28 (3.58, 8.56)	5.25 (3.50, 8.04)	*Z* = −0.442	0.658
Gender, *n* (%)				*χ*^2^ = 0.349	0.554
Female	105 (25.49)	71 (24.65)	34 (27.42)		
Male	307 (74.51)	217 (75.35)	90 (72.58)		
Hypertension, *n* (%)				*χ*^2^ = 0.003	0.955
No	157 (38.11)	110 (38.19)	47 (37.90)		
Yes	255 (61.89)	178 (61.81)	77 (62.10)		
Diabetes, *n* (%)				*χ*^2^ = 0.085	0.771
No	303 (73.54)	213 (73.96)	90 (72.58)		
Yes	109 (26.46)	75 (26.04)	34 (27.42)		
Previous stroke, *n* (%)				*χ*^2^ = 1.066	0.302
No	341 (82.77)	242 (84.03)	99 (79.84)		
Yes	71 (17.23)	46 (15.97)	25 (20.16)		
AF, *n* (%)				*χ*^2^ = 0.140	0.708
No	348 (84.47)	242 (84.03)	106 (85.48)		
Yes	64 (15.53)	46 (15.97)	18 (14.52)		
Smoking, *n* (%)				*χ*^2^ = 0.406	0.524
No	262 (63.59)	186 (64.58)	76 (61.29)		
Yes	150 (36.41)	102 (35.42)	48 (38.71)		
Drinking, *n* (%)				*χ*^2^ = 0.368	0.544
No	333 (80.83)	235 (81.60)	98 (79.03)		
Yes	79 (19.17)	53 (18.40)	26 (20.97)		
Postoperative 48 h fever, *n* (%)				*χ*^2^ = 0.508	0.476
No	250 (60.68)	178 (61.81)	72 (58.06)		
Yes	162 (39.32)	110 (38.19)	52 (41.94)		
Dysphagia, *n* (%)				*χ*^2^ = 0.001	0.975
No	272 (66.02)	190 (65.97)	82 (66.13)		
Yes	140 (33.98)	98 (34.03)	42 (33.87)		
TOAST, *n* (%)				*χ*^2^ = 7.709	0.103
LAA	294 (71.36)	210 (72.92)	84 (67.74)		
CE	60 (14.56)	38 (13.19)	22 (17.74)		
SAO	12 (2.91)	11 (3.82)	1 (0.81)		
SOE	35 (8.5)	20 (6.94)	15 (12.10)		
SUE	11 (2.67)	9 (3.12)	2 (1.61)		
Fazekas, *n* (%)				*χ*^2^ = 0.911	0.823
0 (no lesion)	126 (30.58)	89 (30.90)	37 (29.84)		
1 (mild lesion)	187 (45.39)	127 (44.10)	60 (48.39)		
2 (Moderate lesion)	69 (16.75)	51 (17.71)	18 (14.52)		
3 (severe lesion)	30 (7.28)	21 (7.29)	9 (7.26)		
Infarction site, *n* (%)				*χ*^2^ = 1.306	0.253
Anterior circulation	336 (81.55)	239 (82.99)	97 (78.23)		
Posterior circulation	76 (18.45)	49 (17.01)	27 (21.77)		
Admission mRS, *n* (%)				*χ*^2^ = 0.984	0.321
0–2 (functional independence)	103 (25)	68 (23.61)	35 (28.23)		
3–6 (functional dependence)	309 (75)	220 (76.39)	89 (71.77)		

IQR, interquartile range; NIHSS, National Institute of Health Stroke Scale; GCS, Glasgow Coma Scale; ASPECTS, Alberta Stroke Program Early CT Score; SBP, systolic blood pressure; DBP, diastolic blood pressure; SHR, stress hyperglycemia ratio; TyG, triglyceride-glucose index; CRP, C-reactive protein; NLR, neutrophil-to-lymphocyte ratio; SII, systemic immune-inflammatory index; SIRI, systemic inflammatory response index; PLR, platelet-to-lymphocyte ratio; AF, atrial fibrillation; TOAST, Trial of Org 10,172 in Acute Stroke Treatment; LAA, large-artery atherosclerosis; CE, cardioembolism; SAO, small artery occlusion; SOE, stroke of other determined etiology; SUE, stroke of undetermined etiology; mRS, modified Rankin Scale; Fazekas, Fazekas scale.

### 3.3 Selection of predictive factors for HP

**Figure 2:**
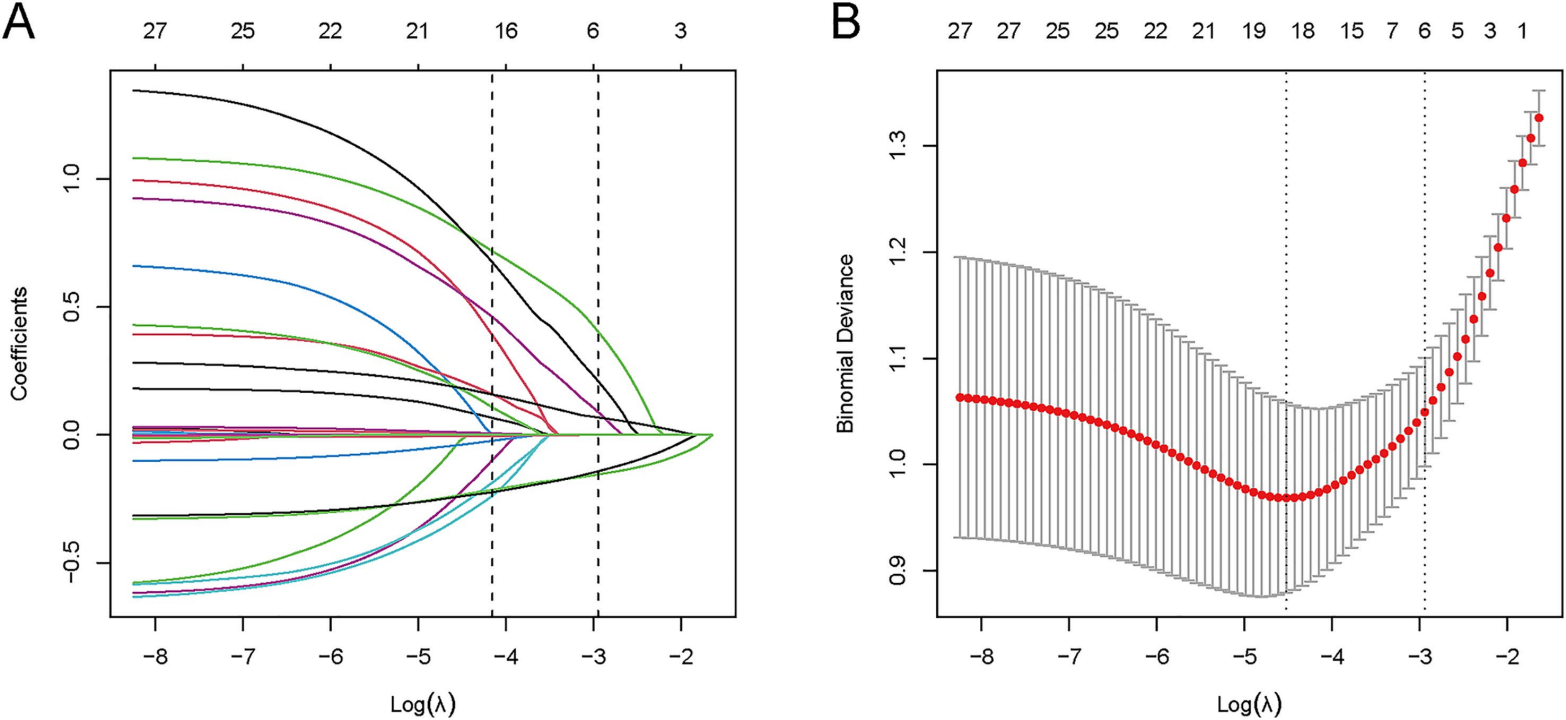
LASSO variable selection process for 28 candidate predictors. **(A)** Coefficient profile plot: LASSO coefficients of 28 features vs. log(*λ*); vertical dashed line indicates λ₁se (λ = 0.053), retaining 6 variables with non-zero coefficients. **(B)** Binomial deviance plot: Mean deviance (*y*-axis) vs. log(λ) (*x*-axis); vertical dashed line marks λ₁se (*λ* = 0.053) from 10-fold cross-validation, which balances model fit and parsimony.

LASSO regression was employed to screen 28 candidate variables, identifying predictive factors with non-zero coefficients (Figures 2A,B). Ten-fold cross-validation was performed to select the optimal *λ* value, which balanced model fit and parsimony. Ultimately, λ_1_se (*λ* = 0.053) was determined as the optimal threshold, leading to the identification of 6 predictive variables: postoperative 24 h fever, dysphagia, admission mRS, admission GCS, ASPECTS, and postoperative 24 h NLR.

**Figure 3:**
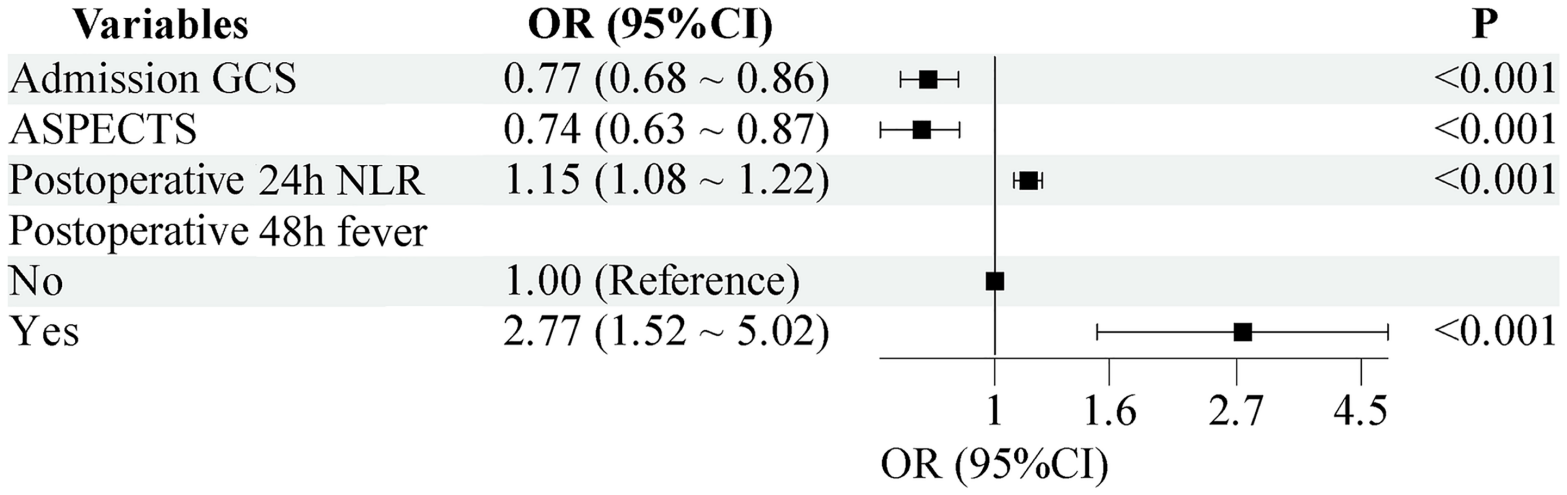
Results of multivariable logistic regression of hypostatic pneumonia after EVT.

Subsequently, these 6 LASSO-selected variables were subjected to multivariate logistic regression analysis, and four meaningful independent predictors were retained for the final model: postoperative 48 h fever, admission GCS, ASPECTS, and postoperative 24 h NLR. The model was constructed with hypostatic pneumonia (HP) occurrence as the dependent variable, targeting AIS-LVO patients who underwent EVT. Results showed that postoperative 48 h fever (OR = 2.77, *p* < 0.001) and postoperative 24 h NLR (OR = 1.15, *p* < 0.001) were independent risk factors for HP, while admission GCS (OR = 0.77, *p* < 0.001) and ASPECTS (OR = 0.74, *p* < 0.001) served as independent protective factors. Specifically, patients with postoperative 48 h fever or elevated postoperative 24 h NLR exhibited a higher risk of developing HP, whereas those with higher admission GCS scores or higher ASPECTS scores had a lower risk, as illustrated in Figure 3.

### 3.4 Construction of the nomogram

**Figure 4:**
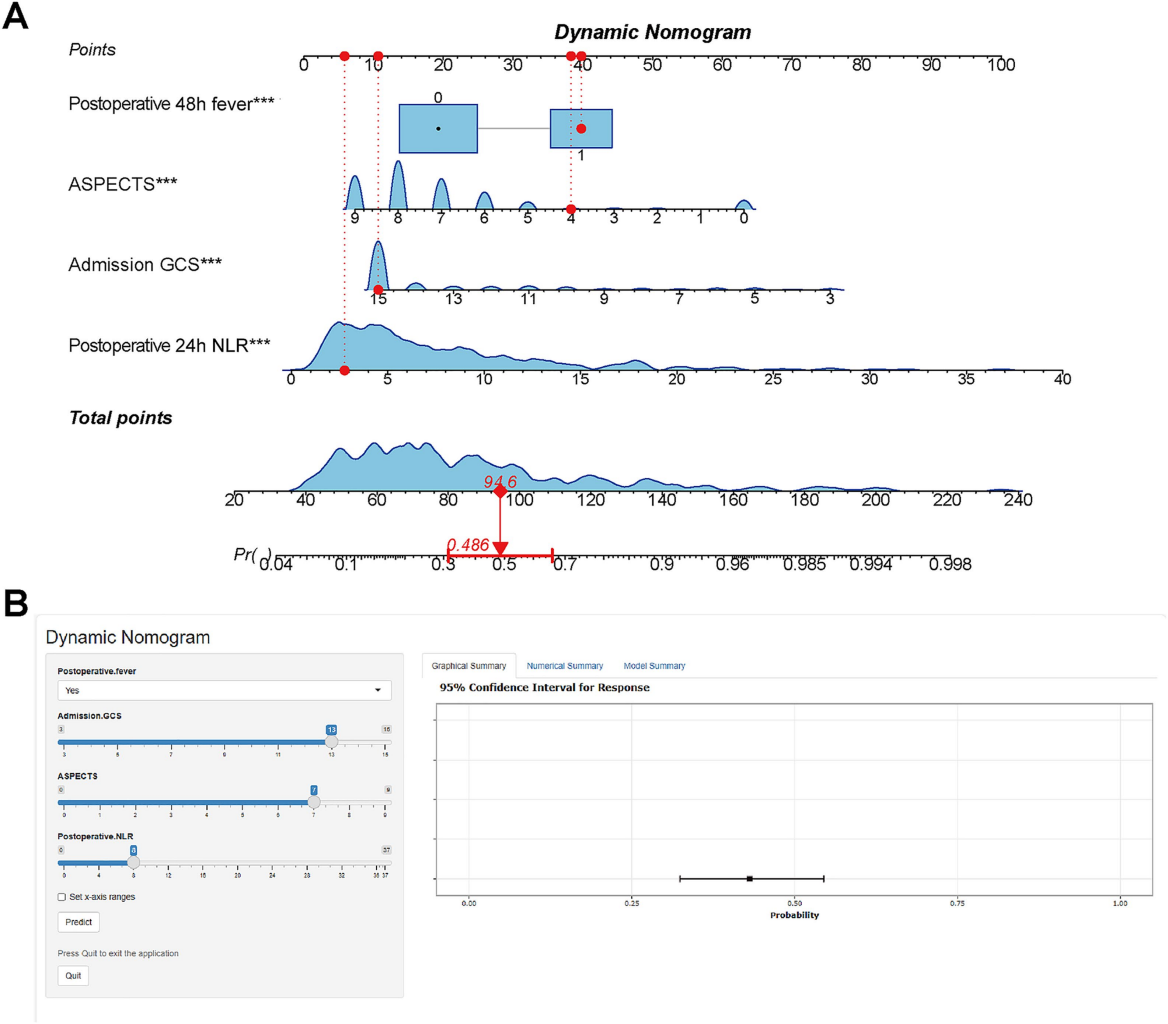
Nomogram for predicting HP risk in EVT patients. **(A)** Nomogram: variables assigned scores; total score estimates HP occurrence probability. **(B)** Web-based tool: Publicly accessible interactive nomogram for clinical use (https://StrokePredMod.shinyapps.io/dynnomapp/).

Based on the results of multivariate logistic regression analysis, we constructed a nomogram for patients with HP after EVT (Figure 4A). Each specific value of the included variables corresponds to a respective score. After summing the scores of all variables to obtain a total score, a vertical line is drawn from this total score to estimate the probability of HP occurrence. To facilitate clinical use by physicians and patients, we have developed a web-based tool for this nomogram (Figure 4B), which is publicly accessible at: https://StrokePredMod.shinyapps.io/dynnomapp/.

### 3.5 ROC validation of predictive model

**Figure 5:**
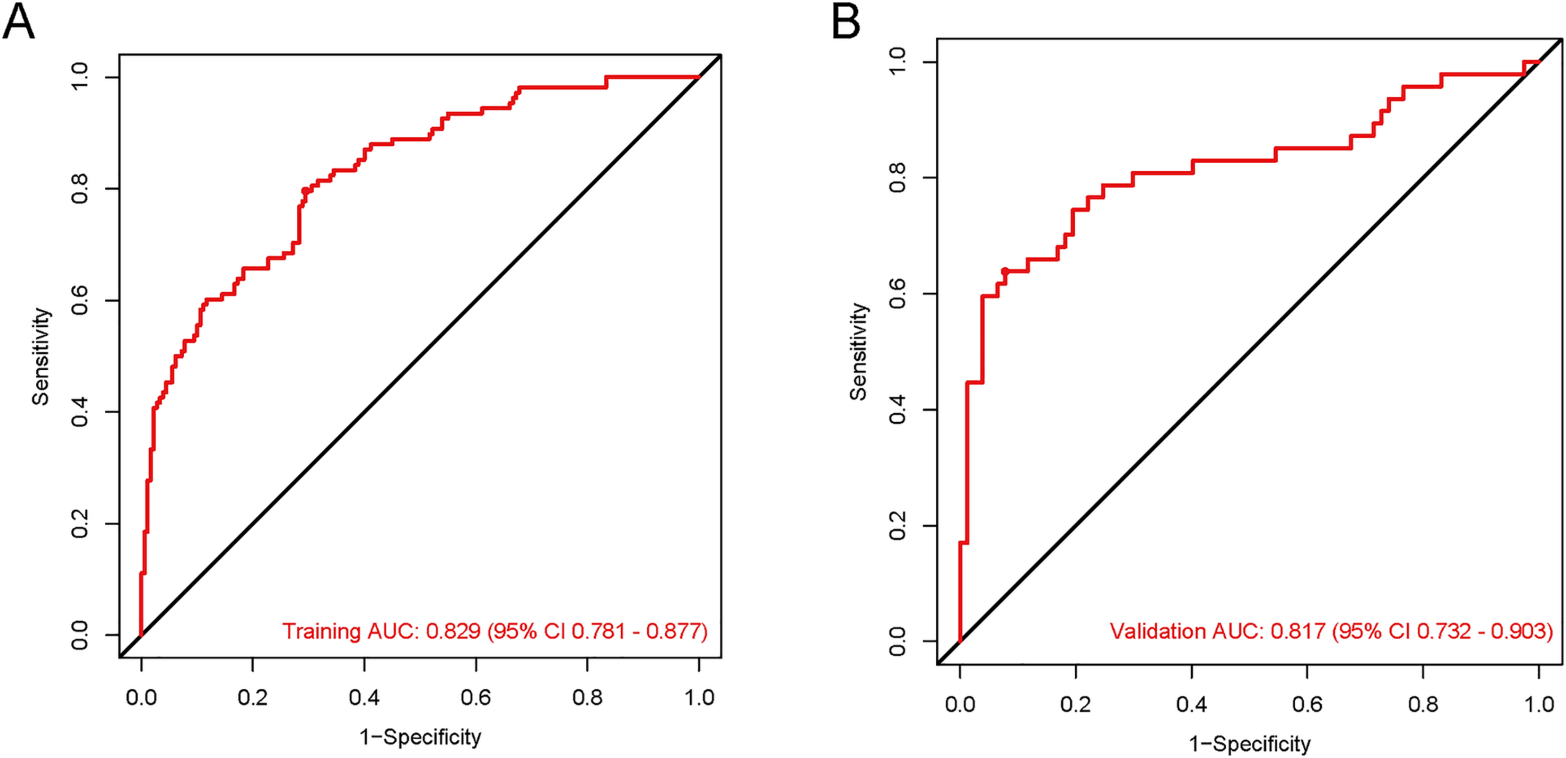
The ROC curves for the HP predictive nomogram in AIS-LVO patients after EVT. In the training cohort **(A)**, the AUROC was 0.829 (95% CI: 0.781–0.877). For the validation cohort **(B)**, the AUROC was 0.817 (95% CI: 0.732–0.903).

To comprehensively assess the model’s discriminatory and diagnostic performance, we report key metrics including the area under the receiver operating characteristic curve (AUC), sensitivity (SEN), specificity (SPE), positive predictive value (PPV), and negative predictive value (NPV) at prespecified thresholds (0.219 for the training cohort and 0.494 for the validation cohort), each with 95% confidence intervals (CIs). As summarized in Table 2, the model achieved an AUC of 0.829 (training set) and 0.817 (validation set), indicating favorable discriminatory ability. At their respective thresholds, the training set showed SEN = 0.796, SPE = 0.706, PPV = 0.639, and NPV = 0.838; the validation set exhibited SEN = 0.638, SPE = 0.922, PPV = 0.802, and NPV = 0.802, all with corresponding 95% CIs. These results collectively demonstrate the model’s consistent and robust diagnostic performance across both cohorts (Figure 5).

**Table 2:** Diagnostic performance indicators of the model based on preset clinical decision thresholds.

Indicator	Training cohort (Cut-off = 0.219, 95% CI)	Validation cohort (Cut-off = 0.494, 95% CI)
AUROC	0.829 (0.781 ~ 0.877)	0.817 (0.732 ~ 0.903)
Sensitivity	0.796 (0.720 ~ 0.872)	0.638 (0.501 ~ 0.776)
Specificity	0.706 (0.639 ~ 0.772)	0.922 (0.862 ~ 0.982)
PPV	0.639 (0.538 ~ 0.739)	0.802 (0.672 ~ 0.932)
NPV	0.838 (0.785 ~ 0.889)	0.802 (0.706 ~ 0.898)

CI, confidence interval; PPV, positive predictive value; NPV, negative predictive value.

### 3.6 Measurement of model performance

To address model overfitting, this study employed the bootstrap optimism correction method and evaluated model performance via 10-fold cross-validation: First, a model was built on the entire original dataset and its apparent performance was recorded. Then, 1,000 bootstrap samples were generated through random sampling with replacement from the original dataset. For each sample, a logistic model was constructed, and its performance was evaluated both on the bootstrap sample and the original dataset. The difference between these two performances was calculated as the “optimism.” After 1,000 iterations, the average optimism was obtained and subtracted from the apparent performance of the original model to derive the optimism-corrected performance. For 10-fold cross-validation, the dataset was randomly divided into 10 equally sized subsets. In each iteration, one subset was used as the test set, while the remaining 9 subsets were combined to form the training set for model construction and evaluation. This process was repeated 10 times to ensure each subset served as the test set exactly once. Finally, the performance metrics were averaged across the 10 folds to robustly estimate the model’s generalization performance and mitigate the risk of overfitting (Table 3).

**Table 3:** Results of cross-validation and bootstrap analysis.

Measures	Fold cross-validation (CI 95%)	Bootstrap optimism-corrected (CI 95%)
AUROC	0.822 (0.769–0.874)	0.927 (0.910–0.952)
Sensitivity	0.898 (0.850–0.946)	0.961 (0.911–1.017)
Specificity	0.596 (0.525–0.667)	0.606 (0.519–0.694)
Accuracy	0.784 (0.746–0.823)	0.828 (0.802–0.858)
Precision	0.786 (0.742–0.829)	0.801 (0.765–0.836)
Recall	0.898 (0.850–0.946)	0.961 (0.911–1.017)

OR, odds ratio; CI, confidence interval.

### 3.7 Model performance and clinical value validation

**Figure 6:**
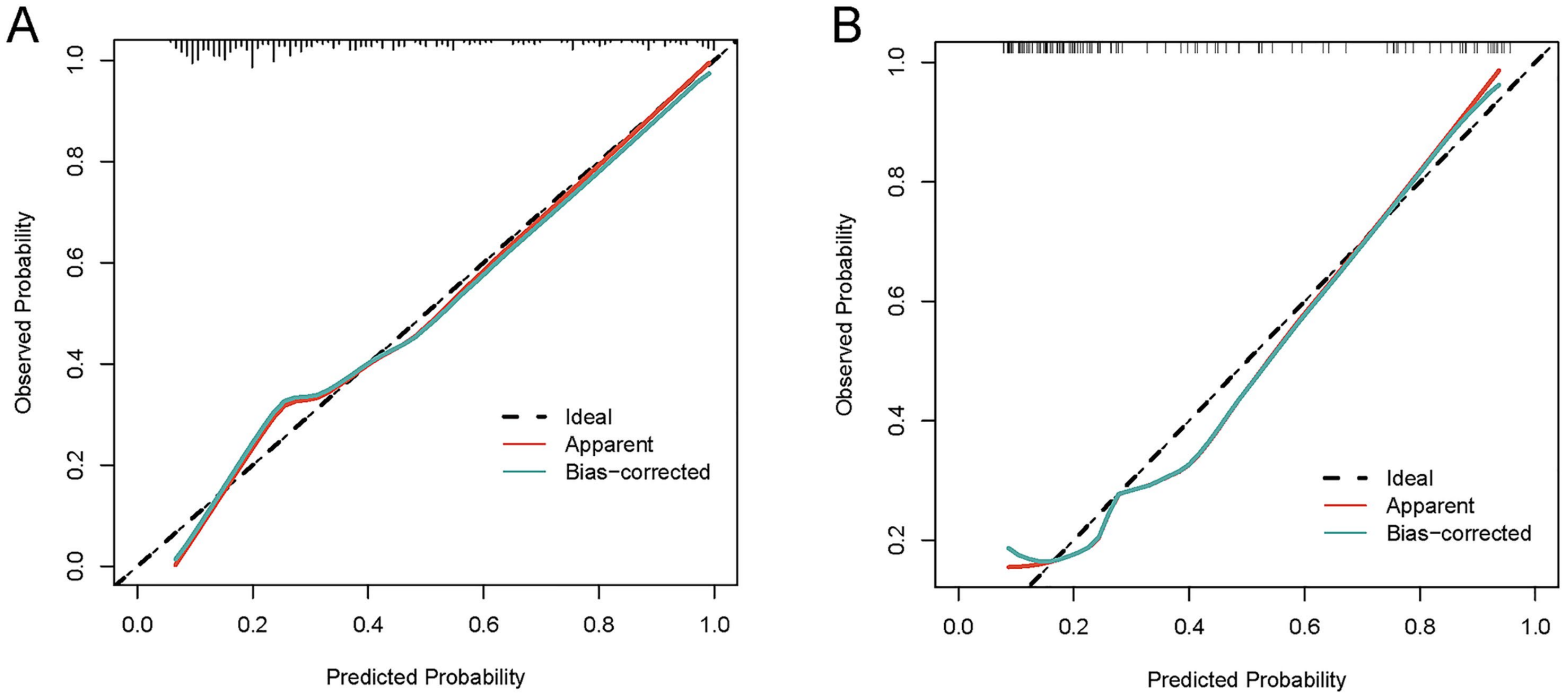
Calibration curves for the HP nomogram. In the training cohort **(A)**, the calibration curve showed good alignment between the predicted probability curve and the ideal reference line. The Hosmer-Lemeshow test yielded *χ*^2^ = 8.9972, degrees of freedom (df) = 8, and *p* = 0.3425; the calibration slope was 0.9995, intercept was −0.042073, and Brier score was 0.1127. In the validation cohort **(B)**, the calibration curve also demonstrated favorable alignment between the predicted probability curve and the ideal reference line. The Hosmer-Lemeshow test yielded *χ*^2^ = 6.4985, df = 8, and *p* = 0.5916; the calibration slope was 1.0582, intercept was 0.074823, and Brier score was 0.1506. These results indicate that the nomogram has reliable calibration performance in both cohorts, with predicted outcomes closely matching actual clinical observations.

**Figure 7:**
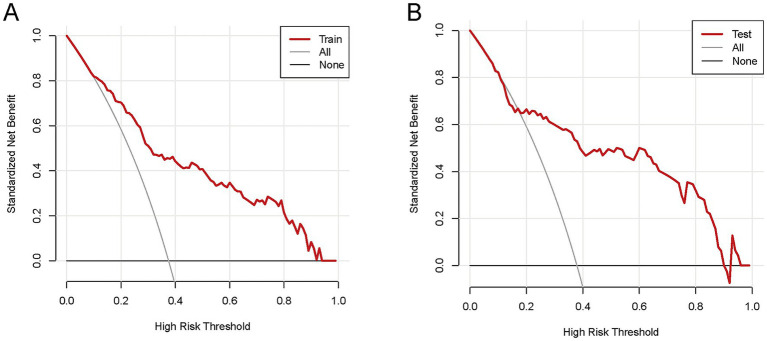
The DCA of the nomogram for predicting HP in AIS-LVO patients after EVT. In the training cohort **(A)**, within the risk threshold range of 9–97%, the model’s net clinical benefit was higher than that of the two extreme strategies (“Treat all patients” and “Treat no patients”). In the validation cohort **(B)**, within the risk threshold range of 11–94%, the model also yielded higher net clinical benefit than these two strategies. These results indicate that the nomogram exhibits reliable clinical utility in both cohorts, providing effective reference for clinical decision-making.

**Figure 8:**
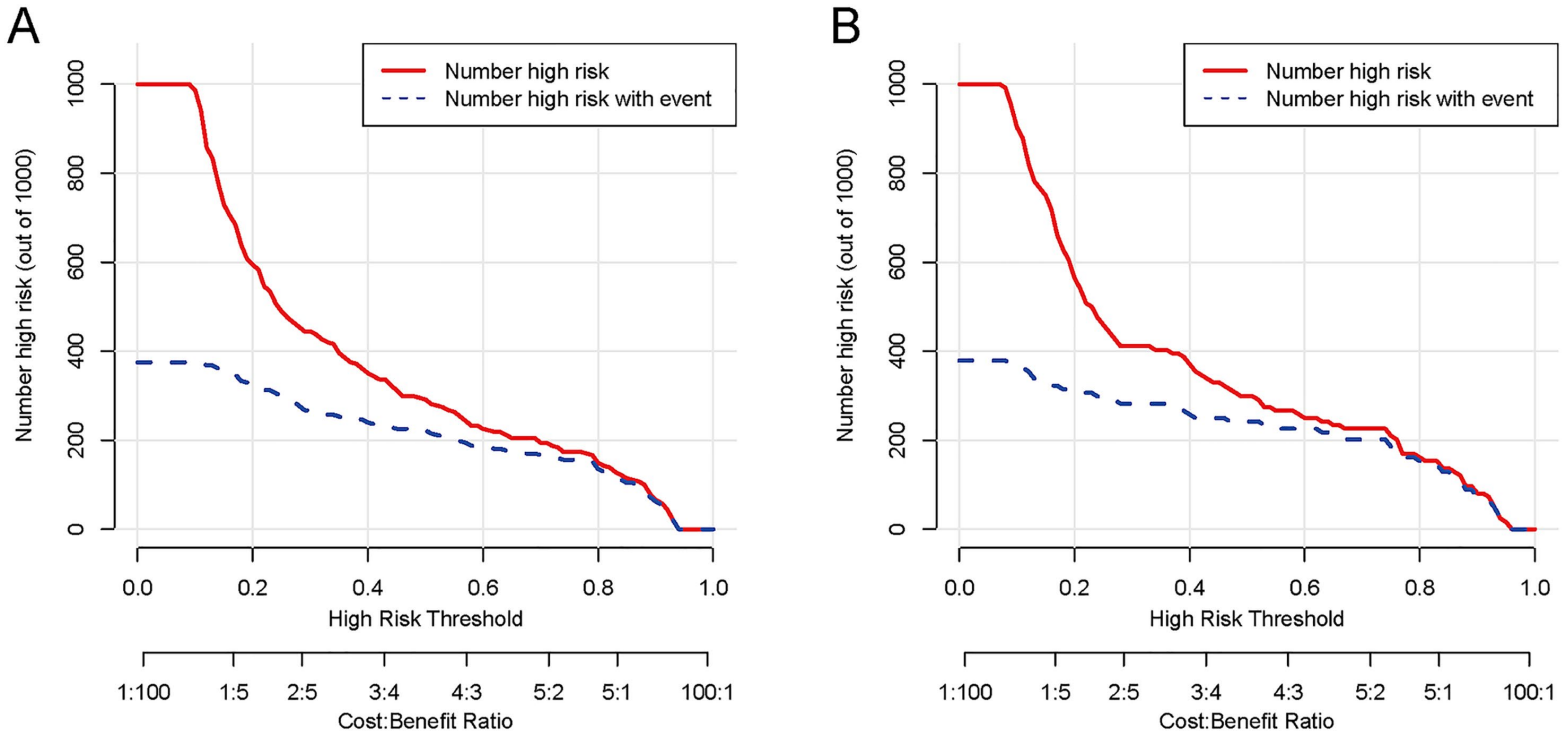
The CIC of the nomogram for predicting HP in AIS-LVO patients after EVT. In the training cohort **(A)**, as the high-risk threshold increases, both the number of model-predicted high-risk patients (red solid line) and actual HP patients (blue dashed line) show a downward trend, with substantial overlap at ~80% threshold (minimizing overprediction). In the validation cohort **(B)**, the curves exhibit a consistent downward trend with the training cohort, overlapping notably at ~80% threshold. These results indicate that the nomogram demonstrates reliable performance across both cohorts, efficiently identifying true HP patients while maintaining a low number needed to treat (NNT).

To comprehensively validate the HP nomogram developed in this study, we systematically evaluated its performance across three critical dimensions: statistical calibration, clinical decision-making utility, and real-world clinical impact. Specifically, this section elaborates on: (1) the model’s ability to achieve agreement between predicted probabilities and actual HP incidence, as demonstrated by calibration curves (Figures 6A,B) and the Hosmer–Lemeshow test; (2) its net clinical benefit across a range of risk thresholds, illustrated via decision curve analysis (DCA, Figures 7A,B); and (3) its efficacy in stratifying and identifying high-risk patients, reflected by clinical impact curves (CIC, Figures 8A,B). Collectively, these findings confirm that the nomogram possesses reliable clinical utility in predicting postoperative HP following EVT in patients with AIS-LVO.

## 4 Discussion

Acute large vessel occlusion ischemic stroke (AIS-LVO) represents a critical neurological emergency with substantial morbidity and mortality (20, 21). Although endovascular therapy (EVT) is a primary treatment modality, postoperative hypostatic pneumonia (HP) can lead to prolonged hospitalization, increased economic burden for patients, and neurological recovery complications (22–24). Our study advances this field by developing and validating the first dedicated prediction model for hypostatic pneumonia specifically in EVT-treated AIS-LVO patients, employing rigorous statistical methodology and demonstrating robust clinical applicability.

Our nomogram identified four independent predictors of HP: (1) lower admission GCS scores, (2) postoperative 48 h fever, (3) elevated postoperative 24 h NLR, and (4) lower ASPECTS scores. These factors collectively reflect the multifactorial pathogenesis of HP, encompassing neurological impairment, systemic inflammation, and environmental exposure-related risks.

The association between impaired consciousness (as quantified by GCS) and pneumonia risk is mechanistically explained by the compromise of airway protective reflexes (25, 26). Our data corroborate previous findings while specifically establishing this relationship in the EVT population. The predictive value of postoperative fever likely reflects both systemic inflammatory response and blood–brain barrier disruption (27), and this pro-inflammatory microenvironment further exacerbates pulmonary secretion accumulation and immunosuppression induced by postoperative immobilization, creating favorable conditions for the development of HP (28). Our study extends current knowledge by demonstrating that early post-EVT fever carries particular prognostic significance, consistent with recent reports of EVT-specific complications (27, 29, 30).

A key methodological innovation of this study lies in the incorporation of the NLR as a practical and sensitive inflammatory marker for analysis. Unlike traditional laboratory parameters, NLR has been proven to be a more reliable indicator reflecting the balance between innate and adaptive immunity. Multiple studies have demonstrated a correlation between this parameter and the risk of nosocomial infections in critically ill patients (31, 32). NLR has been demonstrated to serve as a reflection of the status of systemic inflammation and immunological response (33). The inflammatory response induces the release of cytokines from immune cells, which leads to the creation of anti-inflammatory signals and the subsequent inhibition of cytokine production. This cascade of events serves to suppress infection and prevent disease advancement (34). Nonetheless, a prolonged inflammatory response has been shown to eventually deplete the immune system (35), thereby reducing systemic immunoreactivity, and suppressing systemic cellular immune responses (36). This, in turn, has been demonstrated to lead to an immediate reduction in peripheral blood lymphocyte subsets. This decrease was referred to as stroke-induced immunosuppression syndrome (SIDS). Our findings support the value of NLR in predicting HP following EVT for AIS-LVO patients: elevated NLR within 24 h postoperatively may reflect the dual effects of stroke-induced early inflammation and subsequent SIRS (17). This biphasic immune response creates a vulnerability window for HP—combined with core HP risk factors such as prolonged bed rest and reduced sputum excretion after EVT, suppressed cellular immunity further impairs the clearance of accumulated pulmonary secretions, while the early pro-inflammatory state lays the microenvironmental foundation for pulmonary infection. Collectively, these factors ultimately significantly increase the risk of HP.

Furthermore, NLR is a practical, accessible biomarker for helping to identify patients at higher risk for blood–brain barrier (BBB) disruption and worse prognosis. After ischemic stroke, there is a marked increase in neutrophil infiltration and a reduction in endothelial cell populations, indicating notable BBB disruption. And neutrophils release damage-associated molecular patterns (DAMPs) and matrix metalloproteinases (MMPs), which induce endothelial cell apoptosis and compromise BBB integrity (37). This, in turn, may lead to greater stroke severity, early neurological deterioration, and poor functional outcomes, while amplifying the systemic inflammatory response, further disrupting immune homeostasis and creating favorable conditions for the development of HP (37–39).

Our study also found that ASPECTS scores were negatively correlated with HP risk. As a standardized scale for assessing early ischemic infarction extent, ASPECTS scores directly reflect the size of the cerebral infarction core. From a pathophysiological perspective, lower ASPECTS scores (typically ≤5) indicate a larger infarction volume, which exacerbates the degree of neurological deficit—consistent with our finding that GCS scores are also an independent predictor (40). Expanded infarction can impair the brainstem swallowing center and cough reflex pathways, leading to dysphagia and decreased sputum excretion capacity, which increases the risk of aspiration and secretion accumulation, thereby elevating pneumonia risk (41–43). Meanwhile, large-area cerebral infarction (characterized by low ASPECTS scores) significantly increases the risk of pulmonary infection by triggering a series of immune responses, including leukocyte activation, pro-inflammatory cytokine release, and endothelial damage (40). The brain-pneumonia association involves immune pathways: low ASPECTS scores are correlated with elevated inflammatory markers (e.g., white blood cells, neutrophils, and NLR), indicating that severe ischemic injury can trigger a systemic inflammatory response and impair pulmonary defense (18, 40). Additionally, stroke-induced immunosuppression syndrome (SIDS), characterized by cell-mediated immune disorders and reduced cytokine production, creates a vulnerable window for bacterial invasion (44). This pathophysiological state of concurrent inflammation and immunosuppression highlights the mediating role of systemic inflammation and immunosuppression between brain injury and pneumonia, collectively forming a key bridge connecting severe brain injury and postoperative HP (45). These mechanisms explain the complementary predictive value of ASPECTS and postoperative NLR for pneumonia risk observed in our study.

## 5 Conclusion

The incidence of HP is significantly higher in AIS-LVO patients after EVT. This risk is associated with several factors: (1) lower admission GCS scores, (2) postoperative 48 h fever, (3) elevated postoperative 24 h NLR, and (4) lower ASPECTS scores. By using the scoring system to identify high-risk patients, clinicians can quickly implement targeted interventions, thereby preventing lung infections and improving patient prognosis.

## 6 Limitations

This study has certain limitations. (1) This study was a single-center retrospective investigation. Restricted by insufficient patient diversity across different clinical settings and populations, it inherently suffers from limited external validity and potential selection bias. Although we adopted two complementary methods—bootstrap optimism correction and 10-fold cross-validation—to reduce overfitting risk and enhance result robustness, and further strengthened internal validation and model stability through 1,000 bootstrap resamplings and coefficient of variation (CV) analysis (thereby improving the rigor of internal validation), external validation using multi-center independent datasets has not been performed. Therefore, future studies should prioritize this work to verify the generalizability of the model beyond the single-center population. (2) Due to the retrospective design, continuous dynamic follow-up data of laboratory indicators were unavailable. Although we minimized potential temporal ambiguity bias by strictly defining detection time points (e.g., NLR measured within 24 h after EVT) and excluding patients with preoperative baseline infections, the lack of longitudinal data still limits the evaluation of dynamic inflammatory responses. Additionally, inherent causal biases in observational data (such as unmeasured confounding variables or residual selection bias) cannot be completely eliminated, which may affect the validity of causal inference between predictors and HP risk. (3) Owing to limitations in retrospective data collection, key anatomical variables related to stroke prognosis and infection risk [e.g., involvement of vascular branches, collateral circulation status (e.g., ASITN/SIR grading)] were not included in the model. Furthermore, this study only focused on the short-term incidence of HP after EVT, and the long-term predictive performance of the model has not been evaluated. Future prospective multi-center studies should systematically incorporate anatomical and dynamic inflammatory indicators, extend the follow-up duration, and validate the model in diverse populations to further improve its completeness and predictive efficacy.

## Data Availability

The raw data supporting the conclusions of this article will be made available by the authors, without undue reservation.

## Supplementary material

SUPPLEMENTARY MATERIAL 1 Comparison of ROC values before and after data imputation in sensitivity analysis.
