# Supplementary material for: Development and validation of a prognostic nomogram for predicting hypostatic pneumonia risk in large vessel occlusion stroke after endovascular therapy patients
Source: Front Neurol. 2026 Jan 7;16:1654147. doi: 10.3389/fneur.2025.1654147 (PMC12819305; doi:10.3389/fneur.2025.1654147)
Supplement: SUPPLEMENTARY MATERIAL 1 — Comparison of ROC values before and after data imputation in sensitivity analysis. [file Table_1.DOCX]

**Supplementary Material 2: Results of multicollinearity test**

| Variables | 1/VIF | VIF |
| --- | --- | --- |
| Admission NIHSS | 0.782 | 1.279 |
| Admission GCS | 0.741 | 1.350 |
| Admission mRs | 0.822 | 1.216 |
| Admission Sbp | 0.489 | 2.044 |
| Admission Dbp | 0.508 | 1.970 |
| Postop 24h SHR | 0.857 | 1.167 |
| Postop 24h TyG | 0.739 | 1.354 |
| Postop 24h CRP | 0.865 | 1.157 |
| Postop 24h SII | 0.152 | 6.574 |
| Postop 24h SIRI | 0.618 | 1.617 |
| Postop 24h NLR | 0.486 | 2.058 |
| Postop 24h PLR | 0.212 | 4.709 |
| Postop 48h Fever | 0.879 | 1.138 |
| Age | 0.526 | 1.901 |
| Gender | 0.702 | 1.424 |
| Diabetes | 0.781 | 1.281 |
| Hypertension | 0.770 | 1.299 |
| Atrial fibrillation | 0.789 | 1.267 |
| Prior stroke | 0.879 | 1.138 |
| Smoking | 0.638 | 1.568 |
| Alcohol drinking | 0.720 | 1.389 |
| Dysphagia | 0.832 | 1.202 |
| TOAST | 0.840 | 1.190 |
| Fazekas | 0.637 | 1.569 |
| Operation Time | 0.912 | 1.096 |
| Symptom Onset to Puncture | 0.813 | 1.230 |
| Infarction Site | 0.914 | 1.094 |
| ASPECTS | 0.823 | 1.215 |

**Abbreviations:**  VIF,Variance Inflation Factor.

**Supplementary Material 2: Multicollinearity test for predictor variables​**​

| Variables | 1/VIF | VIF |
| --- | --- | --- |
| Postop 48h Fever | 0.939 | 1.065 |
| Postop 24h NLR | 0.954 | 1.048 |
| Admissions_GCS | 0.873 | 1.145 |
| ASPECTS | 0.880 | 1.136 |
