# Supplementary figures and images for: Development and validation of a prognostic nomogram for predicting hypostatic pneumonia risk in large vessel occlusion stroke after endovascular therapy patients
Source: Front Neurol. 2026 Jan 7;16:1654147. doi: 10.3389/fneur.2025.1654147 (PMC12819305; doi:10.3389/fneur.2025.1654147)

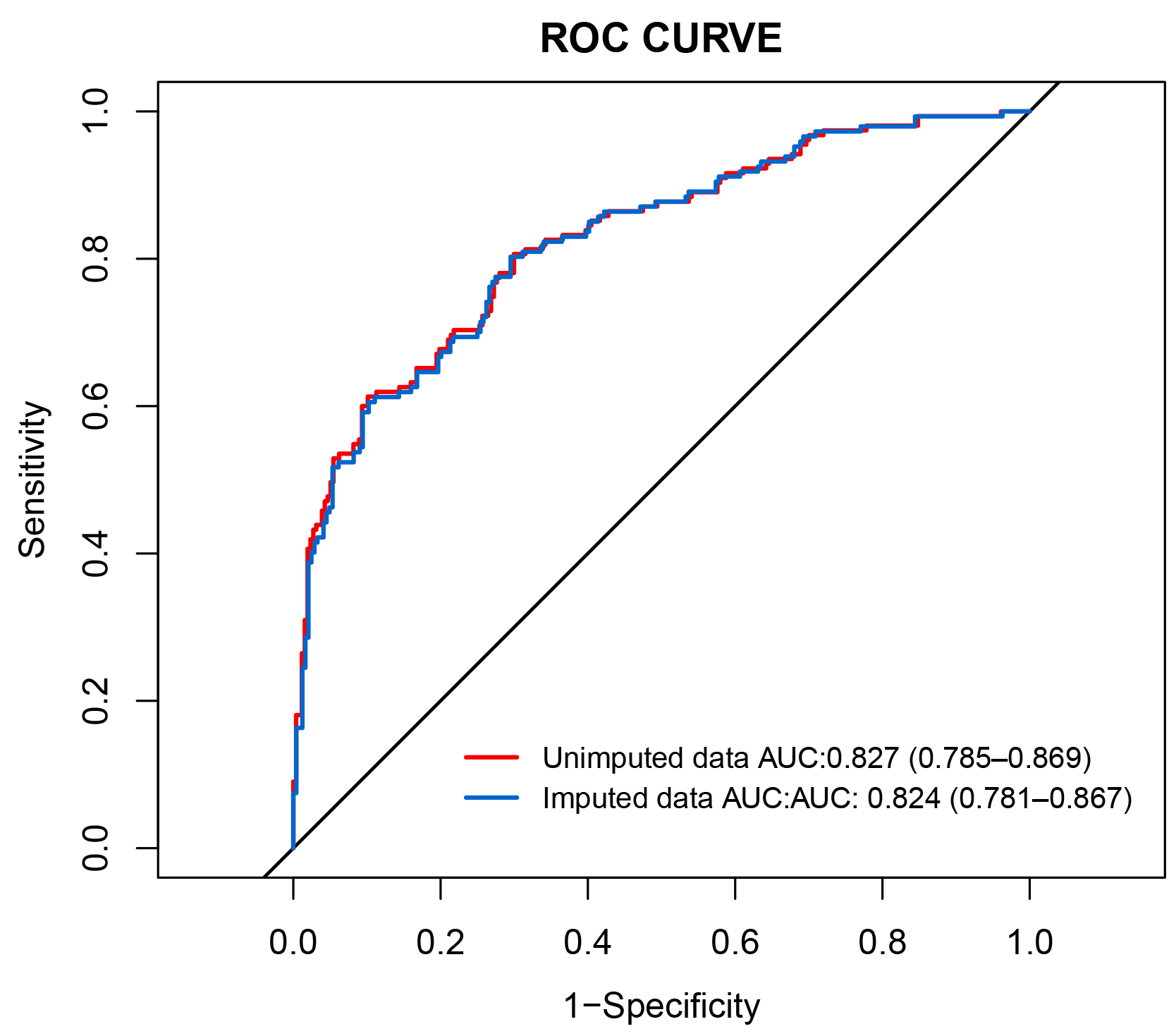

Supplement: Supplementary file 2 [file Image_1.TIF]
